# Lack of evidence of endogenous avian leukosis virus and endogenous avian retrovirus transmission to measles, mumps, and rubella vaccine recipients.

**DOI:** 10.3201/eid0701.010111

**Published:** 2001

**Authors:** A I Hussain, V Shanmugam, W M Switzer, S X Tsang, A Fadly, D Thea, R Helfand, W J Bellini, T M Folks, W Heneine

**Affiliations:** Centers for Disease Control and Prevention, 1600 Clifton Road, Mail Stop G19, Atlanta, GA 30333, USA. whm2@cdc.gov

## Abstract

The identification of endogenous avian leukosis virus (ALV) and endogenous avian retrovirus (EAV) in chick cell-derived measles and mumps vaccines in current use has raised concern about transmission of these retroviruses to vaccine recipients. We used serologic and molecular methods to analyze specimens from 206 recipients of measles, mumps, and rubella (MMR) vaccine for evidence of infection with ALV and EAV. A Western blot assay for detecting antibodies to endogenous ALV was developed and validated. All serum samples were negative for antibodies to endogenous ALV by Western blot analysis. Peripheral blood lymphocyte samples from 100 vaccinees were further tested by polymerase chain reaction for both ALV and EAV proviral sequences; all were negative. Matching serum samples were tested by reverse transcriptase polymerase chain reaction for ALV and EAV RNA, and all 100 samples were negative, providing no evidence of viremia. These findings do not indicate the presence of either ALV or EAV infection in MMR vaccine recipients and provide support for current immunization policies.


### Lack of Evidence of Endogenous Avian Leukosis Virus and Endogenous Avian Retrovirus Transmission to Measles, Mumps, and Rubella Vaccine Recipients

**Althaf I. Hussain,* Vedapuri Shanmugam,* William M. Switzer,* Shirley X. Tsang,* Aly Fadly,† Donald Thea,‡ Rita Helfand,* William J. Bellini,* Thomas M. Folks,* and Walid Heneine***
*Centers for Disease Control and Prevention, Atlanta, Georgia, USA; †Avian Disease and Oncology Laboratory, U.S. Department of Agriculture, East Lansing, Michigan, USA; ‡Harvard Institute for International Development, Cambridge, Massachusetts, USA

The identification of endogenous avian leukosis virus (ALV) and endogenous avian retrovirus (EAV) in chick cell-derived measles and mumps vaccines in current use has raised concern about transmission of these retroviruses to vaccine recipients. We used serologic and molecular methods to analyze specimens from 206 recipients of measles, mumps, and rubella (MMR) vaccine for evidence of infection with ALV and EAV. A Western blot assay for detecting antibodies to endogenous ALV was developed and validated. All serum samples were negative for antibodies to endogenous ALV by Western blot analysis. Peripheral blood lymphocyte samples from 100 vaccinees were further tested by polymerase chain reaction for both ALV and EAV proviral sequences; all were negative. Matching serum samples were tested by reverse transcriptase polymerase chain reaction for ALV and EAV RNA, and all 100 samples were negative, providing no evidence of viremia. These findings do not indicate the presence of either ALV or EAV infection in MMR vaccine recipients and provide support for current immunization policies.

Vaccines effectively reduce and prevent death and disease from many viral infections. However, vaccine production occasionally has been complicated by inadvertent contamination with adventitious agents that may have originated from cell substrates used to propagate vaccine strains. Examples of such contamination include simian virus in early polio vaccines grown on monkey kidney cells and avian leukosis virus (ALV) in yellow fever vaccines propagated in chick embryos (1). Hepatitis B virus has also been identified in yellow fever vaccines produced by using pooled human serum as a stabilizing agent (2). Exposure of vaccine recipients to contaminated vaccines has been associated with effects ranging from benign to demonstrable transmission of infection, with or without subsequent disease (2,3).

Address for correspondence: Walid Heneine, Centers for Disease Control and Prevention, 1600 Clifton Road, Mail Stop G19, Atlanta, GA 30333; fax: 404-639-1174; e-mail: whm2@cdc.gov.

Reverse transcriptase (RT) activity, an indicator of retroviruses, has recently been detected by sensitive polymerase chain reaction (PCR)-based RT assays in currently used vaccines produced in chick embryo fibroblasts or embryonated eggs (4-7). The RT-positive vaccines include measles, mumps, and yellow fever vaccines produced by several manufacturers in Europe and the United States (4,5). RT activity was detected in the vaccines despite strict manufacturing practices requiring that chick embryos and embryo fibroblasts be derived from closed, specific-pathogen-free chicken flocks. Such chickens are screened for known pathogens, including two exogenous avian retroviruses: reticuloendotheliosis virus and ALV (8).

The origin of RT activity in measles vaccines was examined in two recent studies. RT activity in a vaccine manufactured in Europe was associated with particles containing endogenous avian virus (EAV) RNA (6). In the second study, we examined measles vaccines from a U.S. manufacturer and found evidence of both EAV and endogenous ALV (7): we detected particle-associated ALV and EAV-0 RNA sequences in both vaccine and chick embryo fibroblast supernatants and demonstrated neutralization of RT activity in vaccines by anti-ALV RT antibodies. In addition, we observed ALV-like particles by electron microscopy in culture supernatants from chick embryo fibroblasts that had not been inoculated with vaccine viruses (7).

At least six subgroups of ALV (A-E and J) have been identified in chickens on the basis of differences in envelope sequences (9). Only subgroup E viruses are expressed from endogenous sequences that are part of the chicken germ line; all the other subgroups are exogenous. The endogenous ALV sequences are usually referred to as endogenous viral (*ev*) loci. At least 30 *ev* loci have been characterized in various chicken strains (10). Although endogenous ALVs are not known to be pathogenic for chickens, related species of fowl are susceptible to infection with endogenous ALV (11). Disease associations in these cross-species infections have not been fully investigated (11). Exogenous-type ALVs have been shown to cause several neoplastic diseases in infected chickens (9).

Less is known about EAV, which has elements distinct from but closely related to those of the ALV family of endogenous retroviruses. EAV are also present in line-0 chickens (*ev*-negative), which have been bred to have no *ev* proviruses (12). EAV elements exist in at least 50 copies per chicken genome (13).

The observed association of the RT activity of these vaccines with endogenous retroviral particles rather than exogenous retroviruses is consistent with vaccine manufacturing regulations that require exogenous ALV and reticuloendotheliosis virus infections to be eliminated from source chickens. Endogenous retroviral particles are not addressed by current manufacturing guidelines because these particles had not been associated with chick cell-derived vaccines.

The finding of RT activity in all measles vaccine lots from different manufacturers tested suggests that this occurrence is not sporadic and that vaccine recipients may be universally exposed to these retroviral particles (4,5,7,14). Surveillance for infection with ALV/EAV in vaccine recipients is important for evaluating the safety of these vaccines. This surveillance, which was recommended by the World Health Organization in a consultation meeting on RT activity in chicken cell-derived vaccines, is needed for policy decisions regarding the global use of these vaccines (15). We recently reported negative PCR results for ALV and EAV sequences in peripheral blood lymphocytes from 33 measles, mumps, and rubella (MMR) vaccine recipients (7). However, these preliminary results do not fully reflect risks for transmission of ALV and EAV because of the small number of samples analyzed and the lack of testing for antibodies and plasma viremia (7). We have expanded our surveillance for ALV and EAV infection in recipients of MMR vaccines and here report evidence that does not support infection with either ALV or EAV.

### Materials and Methods

### Study Population

The study population was 206 children identified from two cohorts. Samples for 113 of the children were identified from repository serum specimens of the New York City Perinatal HIV Transmission Collaborative Study and Perinatal AIDS Collaborative Transmission Study (PACTS). All 113 children had documented evidence of MMR vaccination; none were infected with HIV-1 (16). The remaining 93 children participated in a study of antibody responses to immunization with the U.S.-manufactured MMR vaccine (17). Of 206 specimens analyzed, 32 (15.5%) were collected 6 to 12 months and 158 (76.7%) 12 to 24 months after the first MMR vaccination. Sixteen (7.8%) samples were collected after the second MMR dose. Peripheral blood lymphocytes samples were available for 100 of 113 children from the PACTS study. All testing was done anonymously with regard to children's identity.

### Endogenous ALV-based Western Blot Assay

The source of antigen for the Western blot assay was the Rous-associated virus type 0 (RAV-0), a prototype endogenous ALV highly related to the endogenous ALV particles found in MMR vaccine (9,10,18). RAV-0 was inoculated into $15B_1$ chick embryo fibroblast cells. Infection with

RAV-0 was monitored by using the ALV antigen test kit (Flockchek, IDEXX, ME) that detects ALV p27 *gag* antigen. Antigen was prepared by lysing $10^6$ cells with 100 µL lysis buffer, followed by 5 minutes each of boiling and sonication. The protein concentration of the lysate was determined with the Pierce protein kit (Rockford, IL).

Electrophoresis was done on 150 µg of either infected or uninfected whole-cell lysates in 10%-20% Tris-HCl gradient SDS-polyacrylamide gels (BioRad, CA) for 2 hours at 70V. Serum samples were diluted 1:50 in blocking buffer. Rabbit anti-avian myeloblastosis virus (AMV, an exogenous strain of ALV) p27 (SPAFAS, Preston, CT) antibody was used as positive control. Anti-rabbit, anti-chicken, and anti-human antibodies conjugated to horseradish peroxidase were used as secondary antibodies for rabbit, chicken, and human plasma samples at 1:6000, 1:3000, and 1:6000 dilutions, respectively. Control human IgG was used as an assay control for anti-human horseradish peroxidase-conjugated secondary antibody.

To validate the Western blot assay, sera from 27 chickens seropositive by virus neutralization assays for ALV (subgroup A) and 34 ALV-seronegative chickens were tested for seroreactivity to ALV antigens by Western blot. In addition, 10 serum samples from chickens infected with reticuloendotheliosis virus were used as specificity controls. Validation on human sera included testing samples from persons infected with human T-cell lymphotropic virus types I and II (HTLV-I and HTLV-II) and HIV types 1 and 2 to assess possible cross-reactivity between ALV and human retroviruses. HTLV- and HIV-negative sera from anonymous blood donors were also included in this validation. All chicken serum samples used for validation of the RAV-0 Western blot assay were also tested on blots containing control antigen from uninfected $15B_1$ chick embryo fibroblasts.

### Proviral DNA PCR

Aliquots of lysates from 150,000 peripheral blood lymphocytes from MMR vaccine recipients were amplified by PCR for ALV *env* and EAV *env*-like sequences by using primers ALVENVF2/ALVENVR2 and EAVENVF10/EAVENVR10, respectively (7). All diagnostic primers used were derived from particle-associated viral sequences identified in the vaccine substrate used to prepare the MMR vaccine (7). Both assays are highly sensitive, with a detection threshold of one copy for EAV PCR assay and 1-10 copies for the ALV PCR assay (7). The PCR reaction conditions included 35 cycles of 95°C for 1 minute, 55°C for 1 minute, and 72°C for 1 minute. PCR products were detected by Southern blot hybridization to the ALV- and EAV-specific $^{32}P$-labeled probes, ALVENVP1 and EAVENVP1, respectively (7).

### Detection of ALV and EAV RNA in Vaccine Recipients

RNA was extracted from serum as described (19). The primers used for the RT reaction were ALVENVR2 and EAVENVR10 for ALV and EAV, respectively. The reaction was carried out at 37°C for 2 hours, followed by 95°C for 5 minutes. RNA extracted from RAV-0-infected $15B_1$ chick embryo fibroblast supernatants was used for positive controls. PCR was carried out as described (7). The ALV and EAV PCR products were detected by Southern blot hybridization with the $^{32}P$-labeled ALVENVP1 and EAVENVP1 probes, respectively.

### Results

### Validation of Western Blot Assay and Criteria for Positivity

The presence of viral proteins was confirmed by the use of antisera raised against whole ALVs (anti-AMV and anti-RAV-0) and against anti-p27 gag protein from AMV (Table). These antisera detected *env* gp85 and gp37 as well as *gag* p27,

Table. Western blot antibody reactivity to the p27 gag protein of the endogenous avian leukosis virus (ALV) in vaccine recipients and other reference chicken and human sera

| Sera tested | p27 Positive |
|---|---|
| Chicken sera (n = 61) | |
| ALV infected/antibody positive[a] | 27/27 |
| ALV uninfected/antibody negative[a] | 0/34 |
| REV infected | 0/10 |
| Human sera (n = 68) | |
| Blood donors | 0/60 |
| HIV-1/2 positive | 0/4 |
| HTLV-I/II positive | 0/4 |
| MMR vaccine recipients (n = 206) | |
| 6-12 months[b] | 0/32 |
| 12-30 months[b] | 0/158 |
| 6-12 months[c] | 0/16 |

[a]antibody reactivity to ALV determined by virus neutralization assays.
[b]samples collected after first MMR vaccination.
[c]samples collected after second MMR vaccination.
REV = reticuloendotheliosis virus; HIV-1/2 = human immunodeficiency virus type 1 or 2; HTLV-I/II = human T-cell lymphotropic virus type 1 or 2; MMR = measles, mumps and rubella p19, p15, and p12 proteins (data not shown). All 27 ALV-infected and neutralization antibody-positive chicken sera reacted strongly to RAV-0 p27, while negative control sera from both uninfected chickens and reticuloendotheliosis virus-infected chickens showed no reactivity to p27 (Figure 1a). These data support the use of p27 reactivity as a marker for ALV seropositivity. Similarly, negative results were seen with samples from 60 human blood donors. No cross-reactivity was observed between RAV-0 p27 *gag* protein and antibodies against HIV-1, HIV-2, HTLV-I, and HTLV-II (data not shown).

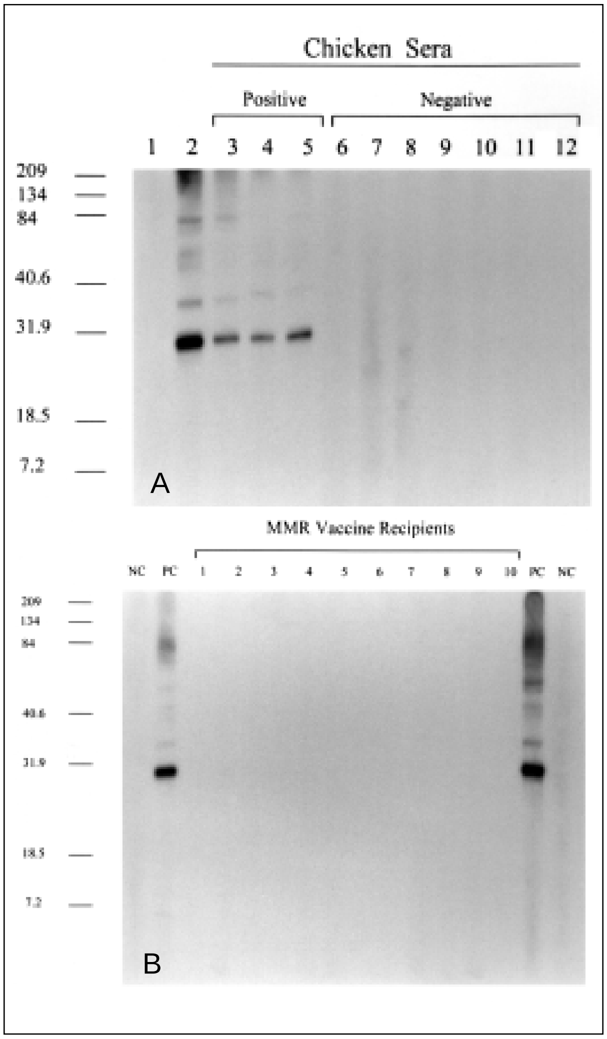

Figure 1. Western blot antibody reactivity of chicken sera to endogenous avian leukosis virus (ALV) (Rous-associated virus 0) antigen. a) Lane 1, negative control chicken serum; Lane 2, positive control anti-p27 ALV gag antiserum; lanes 3-5, sera from ALV antibody-positive chickens; lanes 6-12, sera from ALV-negative, antibody-negative chickens. b) NC, negative control human sera; PC, positive control: anti-p27 ALV gag antiserum; lanes 1-10, sera from measles mumps rubella vaccine recipients.

### Lack of Evidence of Seroreactivity to ALV

Serum samples from all 206 MMR vaccine recipients were negative by Western blot (Table). These samples included those of the 16 children who had received two doses of MMR vaccine (Figure 1b). No seroreactivity to any viral proteins, including p27, was observed.

### Lack of Evidence of ALV and EAV Sequences

All 100 peripheral blood lymphocyte samples were negative for both ALV and EAV DNA sequences by PCR analysis (Figure 2). Of the 100 samples from the PACTS cohort, 33 had been tested previously (7). Similarly, all sera from the 100 children tested negative for both ALV and EAV RNA by RT-PCR (Figure 3). These results indicate absence of both ALV and EAV viremia in these vaccine recipients (Table).

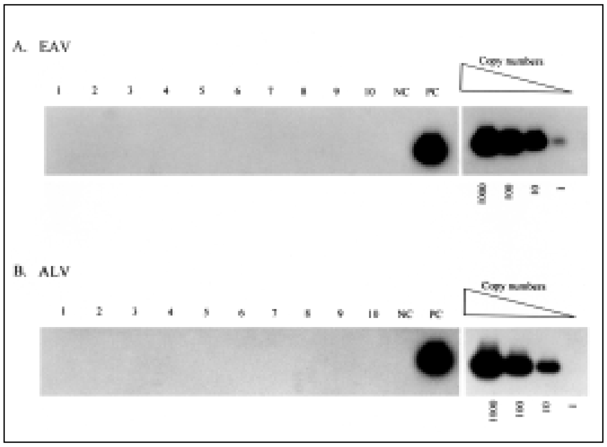

Figure 2. Representative results from polymerase chain reaction analysis of peripheral blood lymphocytes from measles, mumps and rubella (MMR) vaccine recipients for endogenous avian retrovirus (EAV) (A) and avian leukosis virus (ALV) (B) proviral DNA sequences. The detection threshold of known copy numbers of the target sequences is shown in the righthand panels. NC, negative control, uninfected human peripheral blood lymphocytes; PC, positive control, human peripheral blood lymphocytes spiked with 1,000 copies of target sequence; Lanes 1-10, peripheral blood lymphocytes from MMR vaccine recipients.

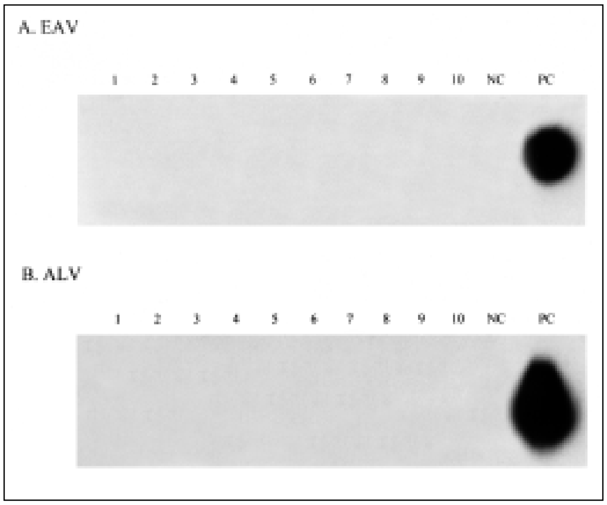

Figure 3. Reverse transcriptase-polymerase chain reaction analysis of sera from measles, mumps and rubella (MMR) vaccine recipients for the presence of avian leukosis virus (ALV) (A) and endogenous avian retrovirus (EAV) (B) RNA. Lanes 1-10, samples from MMR vaccine recipients. NC, negative control, uninfected human serum; PC, positive control, culture supernatant from Rous-associated virus 0 (RAV-0) infected $15B_1$ chick embryo fibroblasts.

### Conclusions

Analysis of MMR vaccines from different manufacturers suggests that vaccine recipients may be universally exposed to endogenous chicken retroviral particles. We sought evidence of persistent ALV and EAV infection in a large number of MMR vaccine recipients, and we were unable to find any evidence of ALV or EAV sequences in peripheral blood lymphocytes, despite the use of highly sensitive PCR assays. Neither did we find evidence of ALV or EAV viremia, since all serum samples tested negative for ALV and EAV RNA by RT-PCR analysis; this finding is of interest because ALV viremia is commonly seen in chickens infected with ALV (18). All 206 serum samples tested by a validated Western blot assay were negative for ALV antibodies, indicating absence of antigenic exposure. These findings differ from those in persons infected with human retroviruses, who usually seroconvert 2 weeks to 6 months after exposure (20,21). The negative serologic data also suggest the low likelihood of nonviremic ALV infection in cells other than peripheral blood lymphocytes, which may not have been detected by PCR testing. Our results overall show no evidence of infection with either ALV or EAV in these vaccine recipients. The lack of transmission of ALV and EAV observed in 16 children who had two MMR vaccinations provides additional reassuring data.

Several factors, including a natural human resistance to infection with endogenous ALV, may explain the lack of transmission of these viruses to MMR vaccine recipients. However, few or no data are available on the ability of endogenous ALV to replicate in human cells. Resistance to endogenous ALV infection may, for instance, be attributed to the absence of a human cell-surface receptor for the virus as well as to other intracellular blocks for ALV replication. A tumor necrosis factor receptor-related protein, referred to as SEAR, has been recently identified as a receptor for endogenous ALV in turkey cells (22). Plasmid-encoded expression of SEAR in human 293 cells can confer susceptibility to infection by endogenous ALV, suggesting that human cells can support endogenous ALV replication if virus entry is achieved (22). Human serum can lyse ALV by complement activation (23); however, this protective mechanism has not been demonstrated for endogenous ALV and EAV particles.

The presence of defective ALV and EAV particles in vaccines may also explain the lack of transmission of these viruses to vaccine recipients. *ev* loci confer a variety of different phenotypes, including infectious or defective particles (9,10,18,24). However, it is not known whether the ALV particles in the vaccine are all defective. The proportion of defective to infectious ALV in different vaccine lots depends on the set of the *ev* loci in the chick embryo fibroblast substrate preparation used for each vaccine lot. Loci associated with noninfectious viruses (*ev*-1, *ev*-3, and *ev*-6) have been identified in a chick embryo fibroblast substrate of a U.S. vaccine manufacturer (7). However, the presence of many loci known to produce infectious ALV-E could not be determined (7).

EAV may represent the predominant retroviral particles in MMR vaccines (6,7). Therefore, our data, which indicate that exposure to EAV particles was not associated with EAV viremia or EAV-infected peripheral blood lymphocytes in vaccine recipients, are important. Confirmation of our molecular results by EAV-specific serologic testing may, however, be necessary. The lack of evidence of transmission of

EAV to vaccinees is likely due to the presence of defective particles. No infectious EAVs have yet been isolated, nor has a full-length intact EAV provirus been identified (25). However, our understanding of the EAV family is limited.

The presence of ALV in chick-cell-derived vaccines is not a new phenomenon; many instances of ALV contamination in yellow fever and measles vaccines have been documented (26,27). However, earlier vaccines had evidence of exogenous rather than endogenous type ALV (27). Available data also suggest lack of transmission of ALV to vaccine recipients (26,28,29). These studies examined 6 adults and 41 children with measles vaccination and 227 yellow fever vaccine recipients (26,27,29), and no evidence was found of neutralizing antibodies to an exogenous ALV. No increase in cancer rate has been found in a study of recipients of exogenous ALV-positive yellow fever vaccine (27).

Despite these reassuring data, the presence of avian retroviral particles in chick embryo fibroblast-derived vaccines raises questions about the suitability of primary chicken cell substrates for vaccine production and the advisability of a change to RT-negative substrates. Chick embryo fibroblasts originating from line 0 chickens could provide substrates that do not express ALV-E; however, such cells may still produce EAV particles (7,12). Obtaining an RT-negative substrate may require a substantial change from primary chicken cells to RT-negative cells from different species, such as immortalized or diploid mammalian cells. Since the cell substrate is critical to the attenuation of live vaccine viruses, any change in the cell substrate could have unpredictable effects on the safety and efficacy of the vaccine and should be approached cautiously.

In conclusion, we found no evidence of either endogenous ALV or EAV infection in recipients of U.S.-made MMR vaccines. Our data indicate that no change is warranted in current U.S. policies for the use of MMR vaccine.

### Acknowledgments

We thank Alison Mawle, Harold Jaffe, and Rafael Harpaz for critical reviews of the manuscript.

This work was supported in part by The National Vaccine Program Office.

Dr. Hussain is a postdoctoral fellow at the Molecular Epidemiology and Zoonoses Section, HIV and Retrovirology Branch, CDC, with research interests in investigating retroviral zoonotic infections.

### References

1. Parkman PD. Safety of biopharmaceuticals: a current perspective. Dev Biol Stand 1996;88:5-7.
2. Norman JE, Gilbert WB, Jay HH, Leonard BS. Mortality follow-up of the 1942 epidemic of hepatitis B in the U.S. Army. Hepatology 1993;18:790-7.
3. Fisher SG, Weber L, Carbone M. Cancer risk associated with simian virus 40 contaminated polio vaccine. Anticancer Res 1999;19:2173-80.
4. Boni J, Stadler J, Reigei F, Shupbach J. Detection of reverse transcriptase activity in live attenuated virus vaccines. Clin Diagn Virol 1996;5:43-53.
5. Robertson JS, Nicolson C, Riley A-M, Bently M, Dunn G, Corcoran T, et al. Assessing the significance of reverse transcriptase activity in chick cell-derived vaccines. Biologicals 1997;25:403-14.
6. Weissmahr RN, Schupbach J, Boni J. Reverse transcriptase activity in chicken embryo culture supernatants is associated with particles containing endogenous avian retrovirus EAV-0 RNA. J Virol 1997;71:3005-12.
7. Tsang SX, Switzer WM, Shanmugam V, Johnson JA, Goldsmith C, Wright A, et al. Evidence of avian leukosis virus subgroup E and endogenous avian virus in measles and mumps vaccines derived from chicken cells: investigation of transmission to vaccine recipients. J Virol 1999;73:5843-51.
8. WHO Expert Committee on Biological Standardization. Requirements for measles, mumps and rubella vaccines and combined vaccines (live). Requirements for biological substances, no. 47. World Health Organ Tech Rep Ser 1994;840:100-207.
9. Coffin JM, Tsichlis PN, Conklin KF, Senior A, Robinson HL. Genomes of endogenous and exogenous avian retroviruses. Virology 1983;126:51-72.
10. Payne LN. Biology of avian retroviruses. In: Levy JA, editor. The retroviridae. New York: Plenum Press; 1992. p. 299-403.
11. Weiss RA, Frisby DP. Are avian endogenous viruses pathogenic? In: Yohn D, Blakeslee IR, editors. Proceedings of the tenth international symposium for comparative research on leukemia and other diseases. Amsterdam: Elsevier;1982. p. 303-11.
12. Resnick RM, Boyce-Jacino MT, Fu Q, Faras AJ. Phylogenetic distribution of the novel avian endogenous provirus family EAV-0. J Virol 1990;64:4640-53.
13. Dunwiddie C, Faras AJ. Presence of reverse transcriptase-related gene sequences in avian cells lacking endogenous avian leukosis viruses. Proc Natl Acad Sci U S A 1985;82:5097-101.
14. Maudru T, Cooperating units, Peden KWC. Analysis of coded panel of licensed vaccines by polymerase chain reaction-based reverse transcriptase assays: a collaborative study. J Clin Virol 1998;11:19-28.

15. WHO. Reverse transcriptase activity in chicken-cell derived vaccine. Wkly Epidemiol Rec 1998;28:209-12.
16. Thomas PA, Weedon J, Krasinski K Abrams E, Shaffer N, Matheson P, et al. Maternal predictors of perinatal human immunodeficiency virus transmission. Pediatr Infect Dis J 1994;13:489-95.
17. Helfand RF, Gary HE, Atkinson WL, Nordin JD, Keyserling HL, Bellini WJ. Decline of measles-specific immunoglobulin M antibodies after primary measles, mumps and rubella vaccination. Clin Diagn Lab Immunol 1998;5:135-8.
18. Crittenden LB. Retroviral elements in the genome of the chicken: implications for poultry genetics and breeding. Crit Rev Poultry Biol 1991;3:73-109.
19. Mulder J, McKinney N, Christopherson C, Sninsky J, Greenfield L, Kwok S. Rapid and simple PCR assay for quantitation of human immunodeficiency virus type 1 RNA in plasma: application to acute retroviral infection. J Clin Microbiol 1994;32:292-300.
20. Lal RB, Heneine W. Testing of human T-lymphotropic virus types I and II: serologic, virologic, and molecular detection. Human T-cell lymphotropic virus type I New York: John Wily and Sons; 1996. p. 167-95.
21. Paul DA, Falk LA, Kessler HA, Chase RM, Blaauw B, Chudwin DS, et al. Correlation of serum HIV antigen and antibody with clinical status in HIV infected patients. J Med Virol 1987;22:357-63.
22. Adkins HB, Brojatsch J, Naughton J, Rolls MM, Pesola JM, Young JA. Identification of a cellular receptor for subgroup E avian leukosis virus. Proc Natl Acad Sci U S A 1997;94:11617-22.
23. Welsh RM, Cooper NR, Jensen FC, Oldstone MBA. Human serum lyses RNA tumor viruses. Nature 1975;257:612-4.
24. Fadly AM. Avian retroviruses. Vet Clin North Am Food Anim Pract 1997;1:71-85.
25. Jacino-Boyce MT, O'Donoghue K, Faras AJ. Multiple complex families of endogenous retroviruses are highly conserved in the genus gallus. J Virol 1992;66:4919-29.
26. Dougherty RM, Harris RJC. Contaminant viruses in two live virus vaccines produced in chicken cells. J Hyg Cambridge 1966;64:1-7.
27. Waters TD, Anderson PS, Beebe GW, Miller RW. Yellow fever vaccination, avian leukosis virus, and cancer risk in man. Science 1972;177:76-77.
28. Levine S, Markham FS. The absence of serologic responses by children and adults to avian leukosis virus in measles vaccine. Archiv fur die gesante Virusforschung 1965;15:305-10.
29. Richman AV, Auliso CG, Jahnes WG, Tauraso NM. Avian leukosis antibody response in individuals given chicken embryo derived vaccines. Proc Soc Exp Biol Med 1972;139:235-7.
